# Present State and Future Prospects of Pediatric Liver Transplantations

**DOI:** 10.31662/jmaj.2018-0009

**Published:** 2018-09-28

**Authors:** Mureo Kasahara, Seisuke Sakamoto, Reiko Horikawa, Akinari Fukuda, Akihiro Umezawa, Yoichi Matsubara, Hitoshi Kato, Takashi Igarashi

**Affiliations:** 1 National Center for Child Health and Development, Tokyo, Japan

**Keywords:** living donor liver transplantation, pediatric liver transplantation, adhesion, small bowel obstruction

## Abstract

Regarding liver transplantations in Japan, with no progress having been made in deceased donor liver transplantations, a living donor liver transplantation performed on a boy with end-stage liver cirrhosis caused by biliary atresia by Nagasue et al. at Shimane University in November 1989 was the first case of its kind. Unlike deceased donor liver transplantations, living donor liver transplantations have two major advantages. First, because organs are donated from healthy adults, it is possible to transplant organs with better viability compared to deceased donor organs, which have been preserved in cold storage for a long time. Second, depending on the recipient’s condition, it is possible to conduct elective surgery at the optimal time. In Japan, the number of annual liver transplantation cases is approximately 400, with the number of annual pediatric liver transplantation cases stable at approximately 120 cases. The patient survival rate of pediatric liver transplantation cases is relatively good at 89.4% over the course of 1 year, 86.8% over 5 years, 84.4% over 10 years, and 80.9% over 20 years.

The liver transplantation program was initiated at the National Center for Child Health and Development, Tokyo, Japan, in November 2005, providing liver transplantation treatment to 510 pediatric patients with end-stage liver disease to date. This article outlines the history of liver transplantations in Japan along with the present state of liver transplantations at the National Center for Child Health and Development.

## History and Present State of Liver Transplantations in Japan

Clinical liver transplantation, a medical treatment with a short history, was initiated in 1963 by TE Starzl et al. in the United States ^(1)^. Although the results were initially unsatisfactory, they remarkably improved because of advances in patient selection, surgical techniques, organ preservation methods, immunosuppressive therapy, and perioperative management, among others. In the United States, approximately 7,500 deceased donor liver transplantations are conducted annually; therefore, this can be considered an established medical treatment for patients with end-stage liver diseases. Liver transplantations in Europe and the United States are centered on deceased donor liver transplantation by harvesting organs from brain-dead or cardiac arrest donors. In Japan, the Organ Transplantation Law took effect in 1997, at which time deceased donor liver transplantation became legally feasible. In 2004, the World Health Organization called on member states as the Istanbul Declaration “to take measures to protect the vulnerable groups from transplant tourism and the sale of tissues and organs, including attention to the wider problem of international trafficking in human tissues and organs.” All countries require a professional framework to govern organ donation and transplantation activities with transparency, as well as a transparent regulatory system that ensures donor and recipient safety and the enforcement of standards and prohibitions on unethical practices. Each country should strive to ensure that programs to prevent organ failure are implemented and provide organs to meet the transplant needs of its residents from donors within its own population ^(2)^. Thereafter, the “Revised Brain Death Bill” in 2010 enabled organ donations from individuals aged 15 years or younger with the consent of their relatives, resulting in a marginally increased tendency for deceased donor organ donation; however, the demand of patients waiting for organ transplantations has not been sufficiently satisfied.

With the background that there has been no progression in deceased donor liver transplantations, liver transplantations in Japan have been centered on living donor liver transplantations using a partial liver from healthy relative donors. The living donor liver transplantation performed on a boy with end-stage liver cirrhosis caused by biliary atresia by Nagasueet al. at Shimane University in 1989 was the first case of its kind in Japan ^(3)^. Living donor liver transplantation is a medical treatment that takes advantage of the following two features: the liver is an anatomically divisible organ, and the liver is a regenerable organ.

Unlike deceased donor liver transplantations, living donor liver transplantations have two major advantages. First, as organs are donated from healthy adults, it is possible to transplant organs with better viability compared to deceased donor organs that have been preserved in cold storage for a long time. It is possible to sufficiently evaluate donors prior to planned transplant surgery, and because the cold storage duration from organ harvesting to transplantation is short because of organ harvesting and transplantation being conducted at the same facility, it is possible to provide a liver with excellent quality blood vessels to be reconstructed and stable conditions for recipients in many cases. Second, depending upon the recipient’ condition, it is possible to conduct elective surgery at the optimal time, even for patients whose condition has suddenly deteriorated or patients who need very urgent treatment. If the consent and safety of a living donor is guaranteed, it is possible to perform liver transplantation as a scheduled surgery with the cooperation of the anesthesiology department, intensive care unit, radiology department, pathology department, nursing department, and administrative department.

In contrast, the disadvantage of living donor liver transplantation is that a liver resection for organ donation is required in healthy living donors despite there being no medical benefits. As donor deaths due to living donor liver transplantation have been reported previously, it is difficult to guarantee the complete safety of living donors because they undergo medically unnecessary liver resection, which is a significantly invasive surgery ^(4)^. In Japan, there is a desire to spread the word and enlighten the population with regard to deceased donor liver transplantations, which are commonly conducted in Europe and the United States.

**Figure 1.**
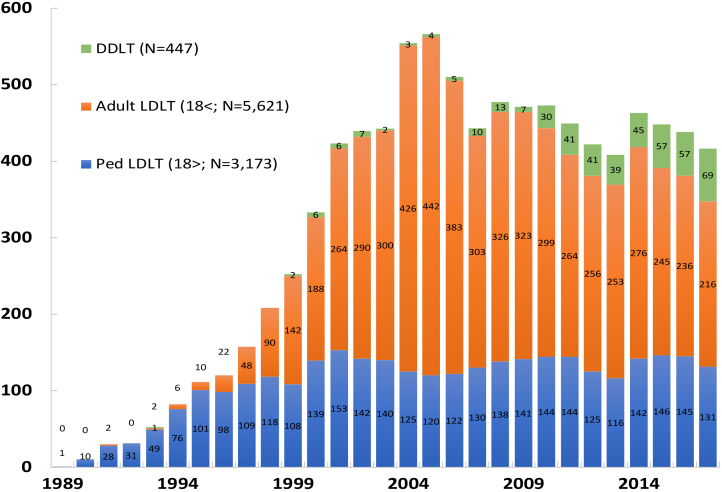
Annual number of liver transplantation cases in Japan (1989–2017; n = 8,347).

In Japan, 8,347 cases of liver transplantation were conducted over a 26-year period from 1989 to the end of 2017 (Figure 1). The number of living donor liver transplantations in children aged under 18 years was 3,173 cases, accounting for 36.8% ^(5)^. In the United States, pediatric liver transplantation cases comprised 7.3% of the total transplantation cases, which is negligible compared with that for adults ^(6)^. This does not mean that pediatric liver transplantations are being proactively performed in Japan. This is because liver transplantations are not sufficiently indicated in adults who can benefit by liver transplantations. In Japan, the number of annual liver transplantation cases indicates a decreasing tendency from 2005. Donor deaths caused by living donor liver transplantations were also reported in Japan on May 5th, 2003 ^(7)^. Adult living donor liver transplantations, which require a larger volume donor liver resection compared with pediatric living donor liver transplantations, are believed to be declining in terms of the number of cases from the viewpoint living donor safety. Nevertheless, the number of deceased donors has doubled since the revised organ transplant law in 2010; the intensification of efforts to reduce living donor morbidity and increase the number of deceased donors have remained important issues in Japan that require attention.

**Figure 2.**
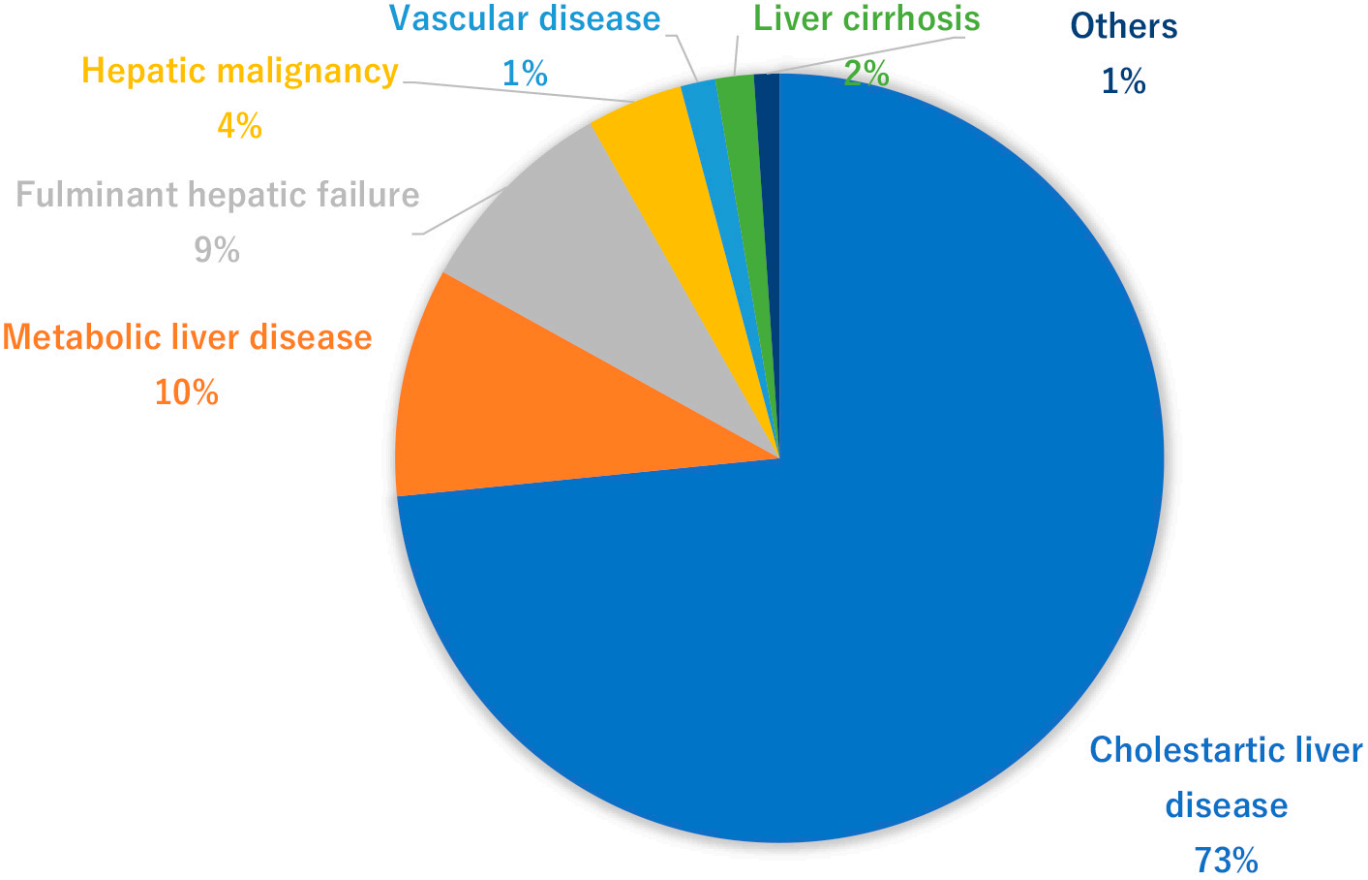
Indication of pediatric liver transplantation in Japan (1989–2017; n = 3,713).

Figure 2 indicates the adaptation diseases from pediatric liver transplantations in Japan ^(5)^. Cholestatic liver diseases represented by biliary atresia account for 73%. Biliary atresia is an unexplained cholestatic liver disease occurring at a frequency of 1/5,000 to 1/19,000 births and is a serious disease leading to cirrhosis from cholestasis. Kasai surgery (portoenterostomy) was developed by Morio Kasai from Tohoku University in the 1950’s, resulting in a dramatic improvement in the short-term survival rate ^(8)^. However, the 20-year survival rate of autologous livers (without liver transplantation) following Kasai surgery is reportedly 49%, suggesting that liver transplantation is required to be performed in childhood for approximately half of the patients as of now ^(9)^. Metabolic liver disease accounts for 10% of the total indication of liver transplantation. Metabolic liver disease is diagnosed by clinical symptoms, such as low activity after birth and vomiting, as well as blood sampling results, such as hyperammonemia and acidosis. If medical treatment for metabolic failure fails, liver transplantation is occasionally indicated. Fulminant hepatitis is a disease leading to liver transplantation indication because of the deterioration of liver functions triggered by drug/virus infections, etc., accounting for approximately 9%. Moreover, liver malignancies represented by hepatoblastoma are also surgically unresectable cases and can be indicated for liver transplantation ^(10)^. The results of pediatric liver transplantation in Japan indicated a 1-year survival rate of 89.4%, a 5-year survival rate of 86.8%, a 10-year survival rate of 84.4%, and a 20-year survival rate of 80.9% in patients, demonstrating relatively better results than those of deceased donor liver transplantations in Europe and the United States. Although it is necessary to take a life-long immunosuppressive drug, pediatric liver transplantation can be considered as an established general medical treatment ^(4)^. However, a decrease in survival rate because of chronic rejection and nonadherence is a major problem in long-term follow-up cases. Going forward, proactive participation of pediatricians in transplantation medicine is desired in Japan.

## Transplantation Medicine at the National Center for Child Health and Development

**Figure 3.**
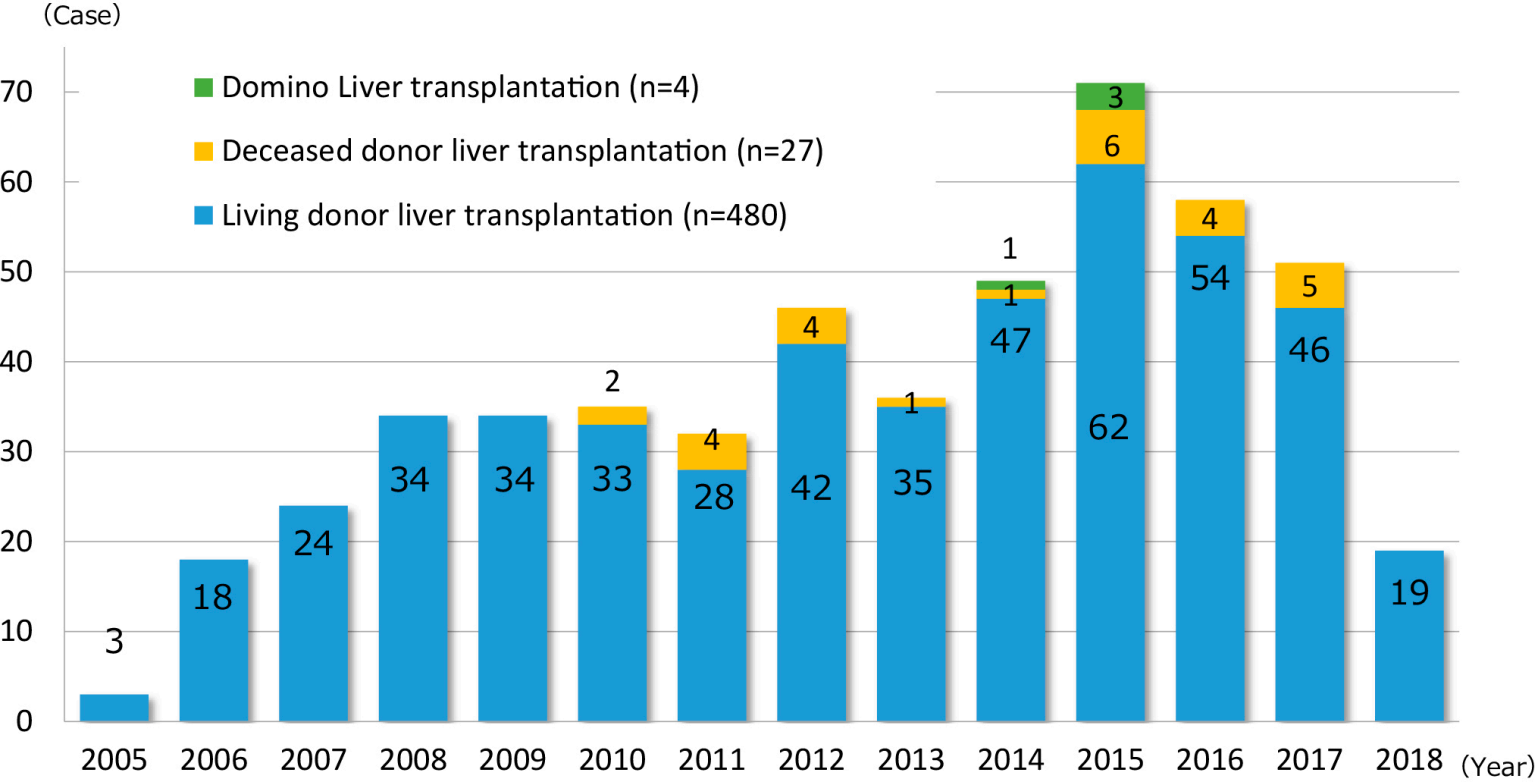
Pediatric liver transplantation in NCCHD (Nov 2005–March 2018; n = 510).

**Figure 4.**
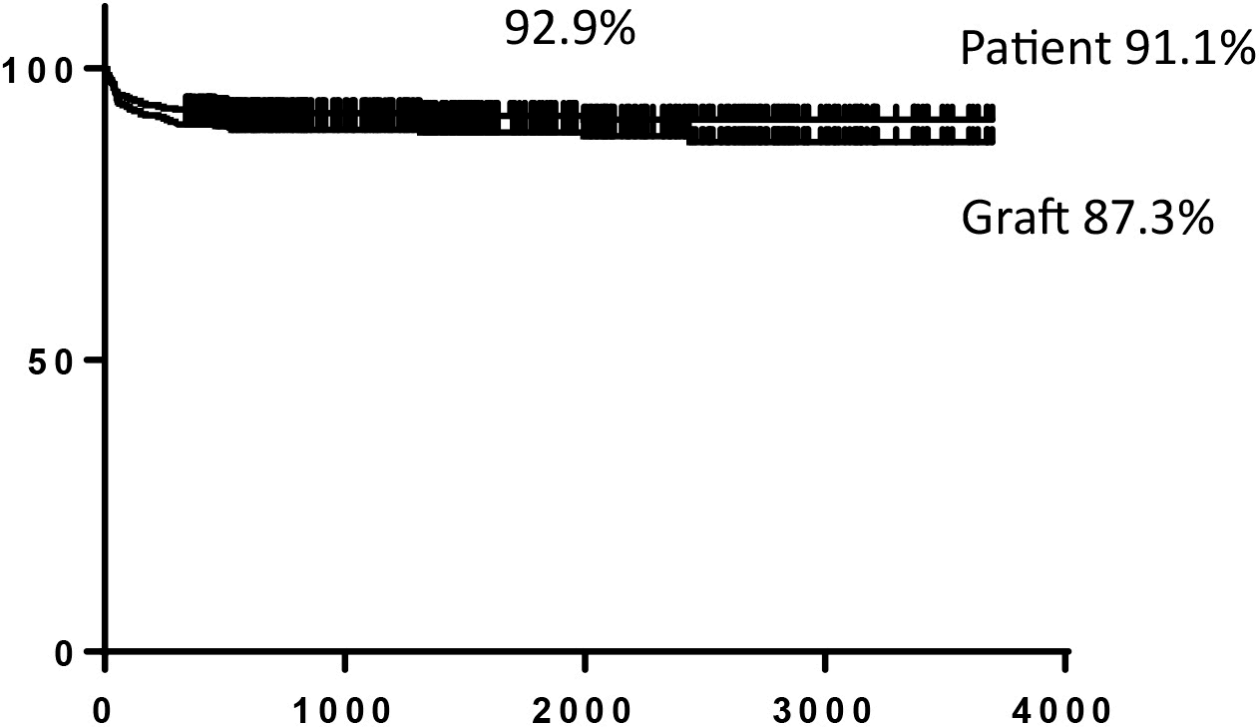
Overall patient and graft survival at the National Center for Child Health and Development.

The National Center for Child Health and Development, Tokyo, Japan, launched the liver transplantation program in 2005, and the Organ Transplantation Center was established in May 2011. We have performed 510 cases of liver transplantation as of May 31, 2018 (Figure 3). The number of pediatric liver transplantation cases per year is 50–70 cases, accounting for approximately 60%–70% of pediatric liver transplantations in Japan. Liver transplantations for cholestatic liver diseases represented by biliary atresia at our center accounted for approximately 50%, which is significantly different from the results of national statistics. This is because our center has handled many serious cases, including metabolic liver diseases (20%) occurring immediately after birth, and fulminant hepatitis (20%) requiring emergency transplantation, as a foundation hospital for pediatric organ transplantation. Although our center treated more serious patients compared with other institutions, we have maintained a relatively high survival rate, with a 10-year survival rate of 92% following transplantation (nationwide average 10-year survival rate: 84.4%), along with no serious complications observed in living donors (Figure 4).

**Figure 5.**
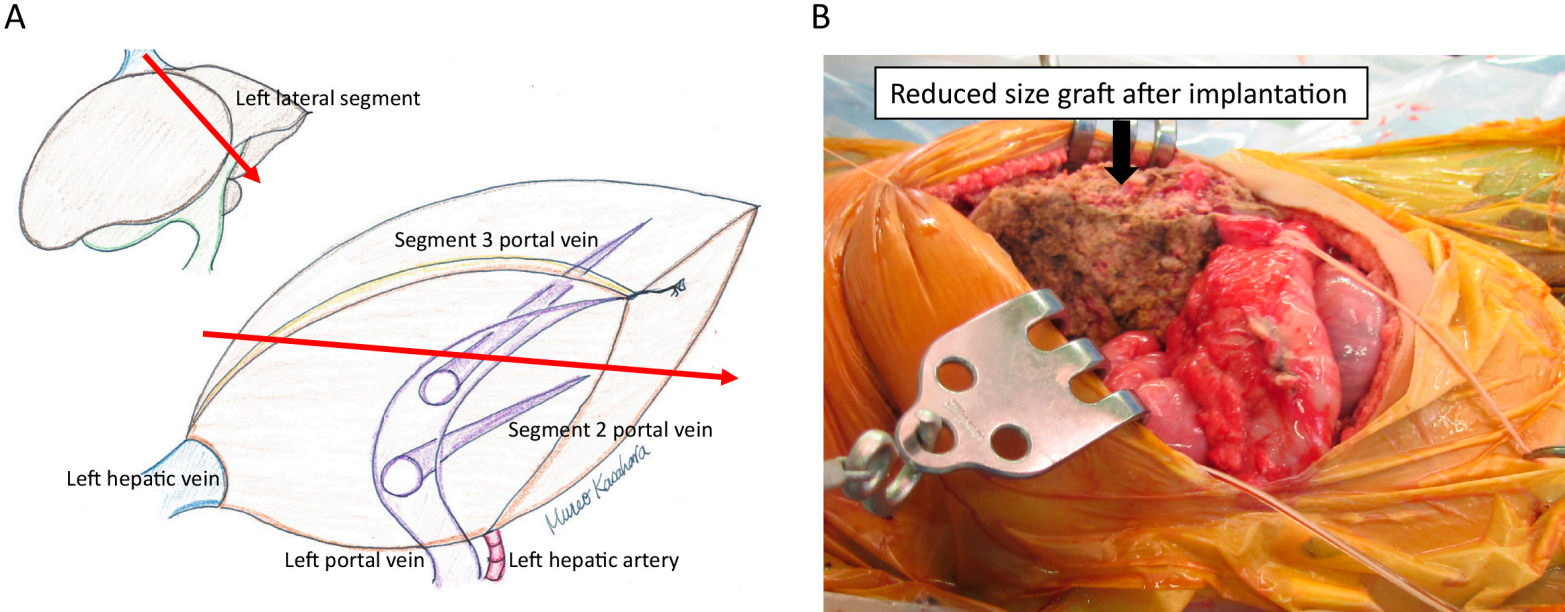
Hyperreduced left lateral segment graft for neonates. A. A schema of the hyperreduced left lateral segment, which can reduce the thickness of the left lateral segment graft. B. After implantation of reduced left lateral segment graft, primary abdominal closure could be possible without compromising blood supply to the graft liver.

Transplanting a whole liver of an adult into the small body of a child is difficult to adapt because it is impossible to maintain sufficient blood flow to the transplanted liver due to compartment syndrome. It has been reported that the optimal transplanted liver weight for children is 2%–4% of their body weight (graft/recipient weight ratio, maximum of 240 g for recipients with a body weight of 6 kg) ^(11)^. In the event of pediatric living donor liver transplantation, the left lateral segment (segment 2 and 3 based on Couinaud anatomical classification) of the liver of adult living donors is used as the transplanted liver; however, the average weight of the left lateral segment of the liver of living liver transplantation donors at our center is 244.3 ± 44.0 g, which is still too large a graft for infants suffering from liver failure with a body weight of 6 kg or less. Therefore, our center has developed a hyperreduced graft from the left lateral segment in which the graft was further surgically reduced and resected. With the development of this new surgical procedure, the results of transplantation for neonatal/infant metabolic liver diseases and fulminant hepatitis remarkably improved ^(12)^ (Figure 5). Liver transplantation treatment can be proactively provided by applying this procedure to fulminant hepatitis in newborns at the 2-kg level whose lives could not be saved in the past, biliary atresia in those with poor weight gain, and metabolic liver diseases occurring in neonates. Moreover, we conducted hepatocyte transplantations in cooperation with the National Center for Child Health and Development Institute by isolating and freezing the surplus liver of living donors as hepatocytes ^(13)^. In some cases of neonatal metabolic liver disease, because the liver is morphologically normal, transplanting normal hepatocytes into the livers of newborns for the purpose of supplementing the enzyme makes it possible to temporarily avoid severe metabolic decompensation. Thus far, we performed hepatocyte transplantations on two newborns with urea cycle disorder who remain well and visit our center on an outpatient basis without neurological complications.

Even in Europe and the United States, where deceased donor liver transplantations are performed as a major organ transplantation procedure, the number of deceased donor organs available for children is very limited. To reduce the number of deaths of pediatric patients with end-stage liver disease waiting for transplantation due to chronic lack of pediatric deceased donor donors, split-liver transplantation, which involves splitting the liver graft from adult deceased donors into the left lateral segment or the left lobe and the right lobe and transplanting each into a child and an adult, respectively, has been developed ^(14)^. The procedure of living donor liver transplantations can also be sufficiently applied to split-liver transplantations. Since the revision of the law in July 2010, our center has registered 120 pediatric patients with end-stage liver disease on the deceased donor liver transplant waiting list and safely performed split-liver transplantations in 20 patients. If the indications are made stricter, even in children, it is possible to expand the donor pool for brain-death liver transplantations by indicating split-liver transplantations.

Our center often receives referred patients with serious high-risk/complications of other organ disorders in the preoperative state, in addition to many cases in which determining the indications for transplantation, management following surgery, or immunosuppressive therapy is difficult. In recent years, the number of patient referrals where it is difficult to manage the perioperative period in transplantations has been increasing. With the aim of further improving treatment results, it is essential to continue clinical research, establish innovative transplantation treatment methods, and develop human resources at the center. In order to spread understanding regarding pediatric organ transplantations, we have opened our doors to other institutions and are accepting many doctor trainees from home and abroad. Particularly in Islamic countries, deceased donor transplantation is difficult for religious reasons, making living donor transplantation the only treatment procedure for patients with end-stage organ failure. The National Center for Child Health and Development proactively accepts doctors/healthcare professionals from Islamic countries and develops medical professionals familiar with transplantation, making it possible to introduce safe living donor liver transplantation to each country. It is already well known as an international pediatric organ transplant institution. To further pursue the safety of pediatric organ transplantation and improve the prognosis thereof, it is important to establish strict indications of transplantation, postoperative management, immunosuppressive therapy, and infection control methods in deceased donor liver transplantation. The National Center for Child Health and Development not only performs clinical organ transplantations but is also involved in joint research, including, with regard to medical hepatocyte transplantation, the development of new immunosuppressive methods, the control of viral infectious diseases following transplantation, clinical application of multivisceral organ transplantation, and regenerative medicine using embryonic stem cells in cooperation with research centers and clinical research and development centers for future children. Moreover, diseases leading to liver transplantation are diverse, many of which are rare diseases that are rarely seen in daily general practices. There are many details to learn from rare diseases, and we are also intensively studying their causes and treatment methods.

## Future of Pediatric Transplantation

Currently, pediatric care in Japan has undergone a significant transformation. Because the system that administers anti-infectious vaccinations has improved and a reduction is expected in the number of hospitalizations because of bacterial meningitis, sepsis, dehydration, and respiratory infections, foundation hospitals for pediatric care must appropriately respond to the necessities of highly advanced medical treatments, including the treatment of newborns with complications, maternal therapy, congenital diseases, pediatric cancer, and organ transplantations.

Organ transplantation is a form of medical care established with the intention of providing precious organs to others. There are very few organ donations from brain-dead patients in our country, and most transplantations for pediatric patients with end-stage organ failure are primarily performed as living donor transplantations with relatives as donors. In the case of pediatric patients with end-stage organ failure who have small bodies, size mismatches with organs from living donors are occasionally observed, rendering it difficult to perform the transplantation. Moreover, there are organ transplantations in which it is impossible to perform living donor transplantations, such as heart transplantations. Because there are many pediatric patients whose lives cannot be saved under the present circumstances of organ transplantation and are dependent on living donors, going forward, enlightenment and promotion of deceased donor organ transplantation will be considered very important issues in Japan. The National Center for Child Health and Development will humbly accumulate such cases so that the lives of more pediatric patients with end-stage organs can be saved. In addition, the center will work to develop new transplantation procedures, study immunosuppressive methods and clinical applications of regenerative medicine, and develop international medical professionals. We hope that transplantations at our center will help further the development of pediatric care/medical care in Japan.

## Article Information

### Conflicts of Interest

None

### Sources of Funding

This work was supported by grants from the Scientific Research Fund of the Ministry of Education and a Research Grant for Immunology, Allergy and Organ Transplant, Rare and Intractable Disease from the Ministry of Health, Labor and Welfare, Japan (grant number 201420032A H27-1, H28-3, H30-1).

### Author Contributions

M.K.: study design, writing of the paper, S.S., A.F., R.H., A.U., Y.M., H.K., T.I.: study design, critical revision of the article for clinical content.
